# Reinstatement of contextual conditioned anxiety in virtual reality and the effects of transcutaneous vagus nerve stimulation in humans

**DOI:** 10.1038/s41598-017-18183-3

**Published:** 2017-12-20

**Authors:** Hannah Genheimer, Marta Andreatta, Esther Asan, Paul Pauli

**Affiliations:** 1 Department of Psychology (Biological Psychology, Clinical Psychology, and Psychotherapy), University of Würzburg, Würzburg, Germany; 2 Institute of Anatomy and Cell Biology, University of Würzburg, Würzburg, Germany; 3 Center of Mental Health, Medical Faculty, University of Würzburg, Würzburg, Germany

## Abstract

Since exposure therapy for anxiety disorders incorporates extinction of contextual anxiety, relapses may be due to reinstatement processes. Animal research demonstrated more stable extinction memory and less anxiety relapse due to vagus nerve stimulation (VNS). We report a valid human three-day context conditioning, extinction and return of anxiety protocol, which we used to examine effects of transcutaneous VNS (tVNS). Seventy-five healthy participants received electric stimuli (unconditioned stimuli, US) during acquisition (Day1) when guided through one virtual office (anxiety context, CTX+) but never in another (safety context, CTX−). During extinction (Day2), participants received tVNS, sham, or no stimulation and revisited both contexts without US delivery. On Day3, participants received three USs for reinstatement followed by a test phase. Successful acquisition, i.e. startle potentiation, lower valence, higher arousal, anxiety and contingency ratings in CTX+ versus CTX−, the disappearance of these effects during extinction, and successful reinstatement indicate validity of this paradigm. Interestingly, we found generalized reinstatement in startle responses and differential reinstatement in valence ratings. Altogether, our protocol serves as valid conditioning paradigm. Reinstatement effects indicate different anxiety networks underlying physiological versus verbal responses. However, tVNS did neither affect extinction nor reinstatement, which asks for validation and improvement of the stimulation protocol.

## Introduction

Recognizing threat and responding appropriately to danger is essential for survival of an organism. Anxiety, also called sustained fear, is an aversive feeling characterized by a diffuse state of apprehension for a possible threat^1^. Anxiety disorders with a lifetime prevalence of 33.7% are the most prevalent mental disorders^2^. Anxiety patients show inappropriate threat associations, as well as impaired fear extinction^3,4^. To investigate associative threat learning conditioning paradigms are used in both rodents^5^ and humans^6,7^, and contextual conditioning is generally used to evoke the diffuse state of anxiety. In such experimental protocols, subjects are exposed to contexts defined by Maren *et al*.^8^ as multisensory, diffuse and continuously present circumstances around an event, which in animal studies may be a cage^9^ or in human studies a long-lasting colour^10^ or picture^11^, or a virtual^12,13^ or a real^14^ room. During acquisition, the subject is repetitively exposed to the context and unconditioned stimuli (US, e.g. mildly painful electric stimuli) are administered without any predicting cue. Consequently, the US and this context become associated, the context evolves into the anxiety context (CTX+) and elicits anxiety responses like freezing^15^ or startle potentiation^16^. In differential conditioning, a second context never paired with an US becomes the safety context (CTX−). In extinction training, participants are repetitively exposed to the contexts again (i.e. CTX+ and CTX−), but no US is administered. This leads to the creation of a new, inhibitory extinction memory trace, which competes with the anxiety memory^17–20^.

As the anxiety memory, i.e. the CTX−-US association, still exists after extinction, anxiety can return^17^. One of the relapse mechanisms is reinstatement, which is the return of conditioned anxiety to the extinguished context after another unexpected presentation of the US^17,21^. While such return of fear in humans is quite well examined in cue conditioning, only few studies exist for contextual conditioning. We recently reported anxiety relapse after reinstatement dependent on state anxiety^22^. Interestingly, high state anxious individuals showed differential reinstatement in terms of potentiated startle responses in CTX+ compared to CTX−, whereas low state anxious individuals showed generalized reinstatement indicated by generally enhanced startle responses^22^.

Exposure therapy, an effective treatment for anxiety disorders, is assumed to induce extinction, strengthen the extinction memory trace and reduce anxiety-like behaviours^23,24^. Some anxiety patients, however, fail to respond to exposure therapy^23,25^ or relapse after treatment which may be due to a strong and intact fear memory or due to deficits in extinction learning^17^. Therefore, studies on the facilitation of extinction learning and/or prevention of return of anxiety may lead to more effective therapies.

Vagus nerve stimulation^26^ (VNS) might be a promising method to activate the crucial brain network involved in the formation and consolidation of extinction memory. Approximately 80% of the vagal fibres are afferent and carry primarily viscerosensory information to the nucleus tractus solitarius (NTS), which transduces the information further into the brain^27^. Studies in experimental animals have documented that activation of vagal afferents, which during an emotional experience may be elicited by peripheral adrenalin binding to vagal β-adrenergic receptors^28^, mediates the release of norepinephrine (NE) in key structures of emotional memory formation including the amygdala, hippocampus and ventromedial prefrontal cortex (vmPFC)^29,30^, either directly via the NTS or indirectly via the locus coeruleus (LC)^31,32^. NE in these forebrain areas plays an important role in memory formation and consolidation, particularly during fear extinction^18,33^. VNS may thus be regarded as a tool to enhance the communication between the periphery and the central nervous system (CNS) during extinction learning^33^. Indeed, VNS-induced facilitation of extinction learning was supported by Peña *et al*.^34^, who performed a conditioning experiment in rats applying electric foot shocks during a 30 s lasting tone. Subsequently, the tone elicited sustained fear. In this single cue conditioning experiment, they demonstrated faster extinction and less return of conditioned freezing in rats which received vagus nerve stimulation via implanted electrodes during extinction compared to a sham stimulated group. In their follow-up study, Peña *et al*.^35^ also demonstrated similar results for the extinction of context conditioned anxiety indicated by decreased freezing behaviour in VNS compared to sham stimulated rats when exposed to the experimental cage.

In humans, stimulation of the vagus via electrodes implanted along the cervical portion of the nerve is an established treatment for medically intractable epilepsy^36^ and is increasingly used as add-on therapy in various psychiatric disorders including depression, dementia and others^37^. Recently, a non-invasive VNS technique has been developed which makes use of the fact that the cymba conchae of the human external ear is innervated exclusively by the auricular branch of the vagus nerve (ABVN)^38^. The ABVN has the same ratio of afferent myelinated A beta axons as the cervical vagus nerve (CVN), which indicates similar effects of transcutaneous vagus nerve stimulation (tVNS) via the ear^39^. Although the ABVN relates somatosensory rather than viscerosensory information to the brainstem, its central projections reach the NTS^40^, and recent evidence documents that transcutaneous stimulation of this area leads to widespread changes in the activation states of the NTS and of other primary and higher-order targets of vagal sensory information in brainstem and forebrain^41^. Burger *et al*.^42^ investigated the effects of tVNS compared to Sham stimulation in healthy humans using a classical cue conditioning paradigm with geometric shapes. They found accelerated fear extinction in US expectancy ratings. Since they did not observe effects of differential physiological conditioning during acquisition, no conclusions for extinction effects could be made on this parameter. A study in humans investigating the effects of tVNS on the extinction of contextual anxiety and relapse is still missing. For this investigation, we modified the context conditioning paradigm in virtual reality used in Glotzbach-Schoon *et al*.^22^ in order to fit it with the stimulation requirements and reinstatement parameters. Additionally, we exchanged the head mounted display used in the Glotzbach-Schoon study^22^ for a Powerwall in the present paradigm. This set up presents the advantage that participants can see their own body which increases feelings of presence (see Methods section).

The objectives of the current study were two-fold: first, to test reliability of the modified virtual reality paradigm for inducing and assessing, both on a physiological and a subjective level, memory consolidation between acquisition, extinction and reinstatement of contextual anxiety; second, to translate the experimental approach of the Pena *et al*. animal studies^34,35^ to the human situation by examining the effects of tVNS on extinction and reinstatement, using carefully controlled conditions with both sham stimulated and non-stimulated controls.

## Results

### Evaluation of stimulation conditions

Participants were assigned randomly to one of three experimental groups: Verum stimulation (VNS), sham stimulation (Sham) and no stimulation (control). In order to check the comparability of all three groups, we assessed participants’ subjective feeling about the stimulation efficacy, their experienced stimulation operability and the subjective pleasantness of the stimulation (valence) after the experiment. Importantly, all groups were similarly convinced about the stimulation efficacy (*F*(2,72) = 2.10, *p* = 0.130, *ƞ*_*p*_^2^ = 0.055; Table 1), however, differed in the evaluation of the experienced operability (*F*(2,72) = 3.51, *p* = 0.035, *ƞ*_*p*_^2^ = 0.089) and valence of the stimulation (*F*(2,72) = 4.13, *p* = 0.020, *ƞ*_*p*_^2^ = 0.103). In comparison to the control group without any stimulation, the VNS group reported a similar operability (*t*(48) = 0.76, *p* = 0.451) and more negative valence (*t*(48) = 2.40, *p* = 0.020). The Sham group compared to the VNS group reported less negative valence of the stimulation (*t*(48) = 2.80, *p* = 0.007).

**Table 1:** Rating of stimulation. Participants were divided into three groups: Verum vagus nerve stimulation (VNS) at the cymba concha of the left ear, sham stimulation (Sham) at the helix and a control group (Control), in which the stimulator was applied to the cymba concha but never switched on. All participants rated their conviction of the stimulation efficacy (0 = not convinced, 10 = very convinced), the operability of the stimulation (0 = stimulation did not work, 10 = stimulation worked well), and the valence of the stimulation (0 = unpleasant, 10 = pleasant). #Difference of VNS and Sham group. ^+^Difference of VNS and Control group. ^~^Difference of Sham and Control group.

	VNS	Sham	Control	statistics
Conviction (SD)	5.68 (0.36)	6.44 (0.43)	5.24 (0.47)	*p* = 0.130
Operability (SD)	7.20 (0.44)	8.36 (0.40)	6.68 (0.53)	*p* = 0.035^*,~^
Valence (SD)	5.20 (0.40)	6.84 (0.43)	6.84 (0.56)	*p* = 0.020^*,#,+^

*p < 0.05.

### Acquisition of conditioned contextual anxiety (Day 1)

**Figure 1:**
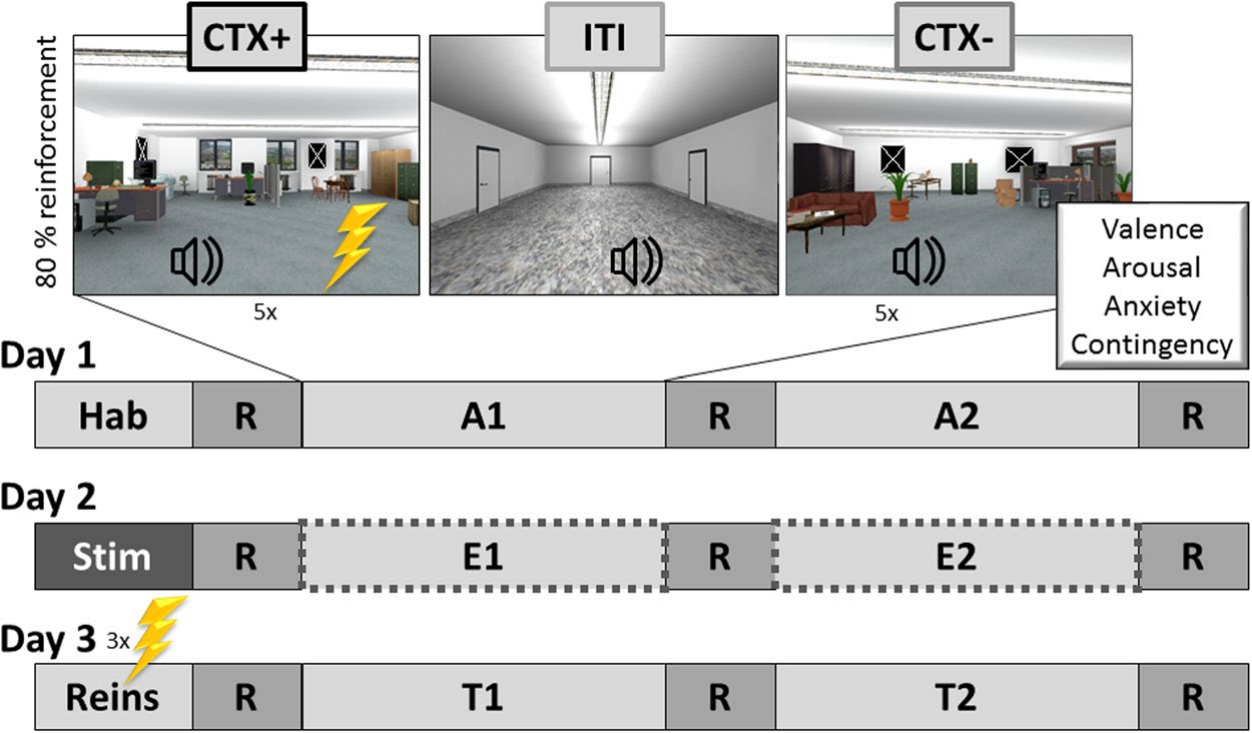
Experimental Design. Depicted are pictures of the three virtual contexts (anxiety context, CTX+; safety context, CTX−; and corridor, inter-trial interval, ITI). On Day 1, the experiment started with the habituation phase (Hab). Anxiety conditioning occurred during acquisition 1 and 2 (A1 and A2). Day 2 began with a 20 min stimulation for the VNS and Sham group. During both extinction phases (E1 and E2), stimulation occurred simultaneously to the stay in one office. No US was presented in this phases. Three US for reinstatement (Reins) were administered at the beginning of Day 3. The subsequent test phases (T1 and T2) were similar to the extinction without stimulation. Ratings of valence, arousal, anxiety and contingency were assessed on each day between phases. Screenshots of the virtual environment were made in house. Wall pictures implemented in VR were taken from IAPS^70^ database and are blackened here.

The experimental paradigm is depicted in Fig. 1. For a detailed description see ‘Experimental Procedure’ in the Methods section. In the habituation phase, participants explored the two virtual offices used in the study, which were connected by a corridor (Inter-Trial-Interval, ITI). During acquisition one virtual office was unpredictably paired with an unconditioned stimulus US (electric shock). This context served as anxiety context (CTX+) whereas the other office, in which the US was never applied, served as safety context (CTX−).

Please note that all statistical analyses of startle, arousal, anxiety and contingency included the factor group, however, no significant main or interaction effects involving this factor were revealed during acquisition.

#### Startle

**Figure 2:**
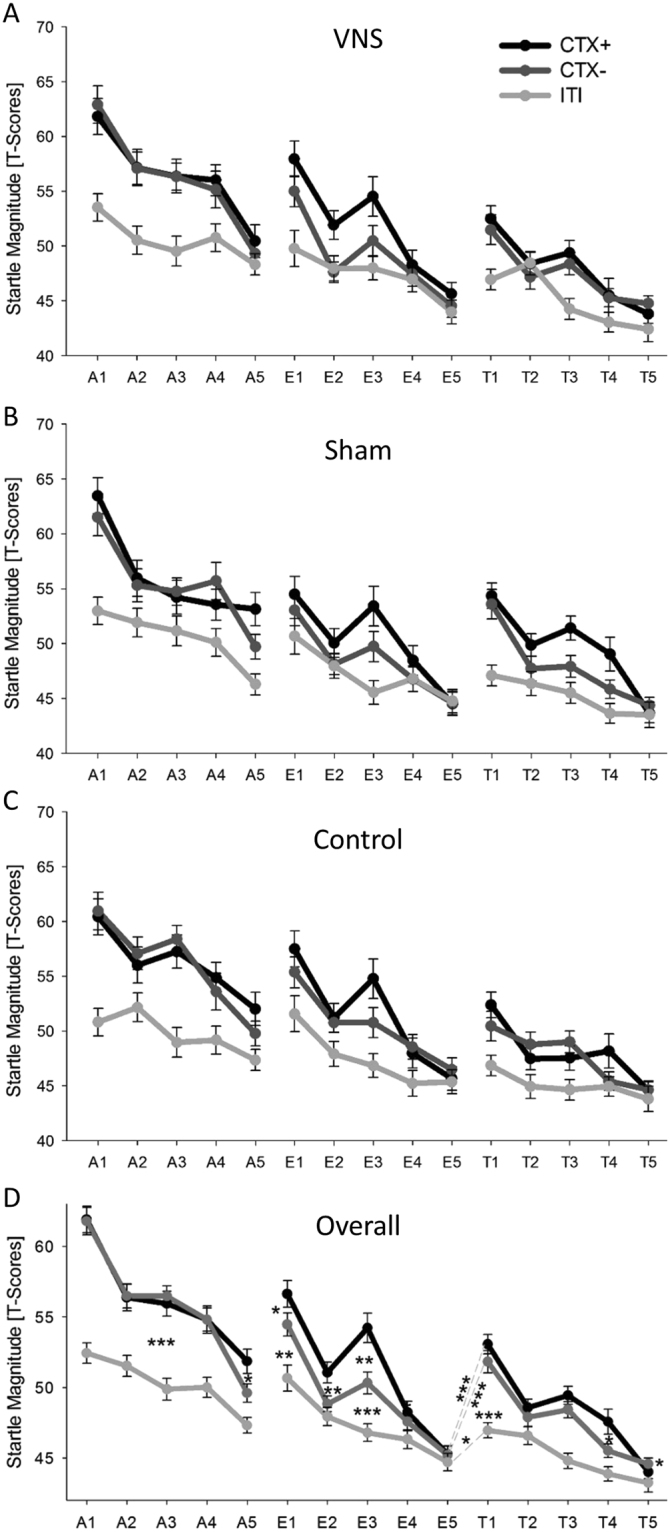
Startle responses. T-scores of the startle magnitude are depicted in the anxiety context (CTX+), safety context (CTX−) and corridor (ITI) separated by acquisition on the first day (A1-A5), extinction on the second day (E1-E5), and reinstatement/test phase on the third day (T1-T5). Each point on the x-axis depicts the mean of 2 trials. (**A**), (**B**) and (**C**) show startle responses of the VNS group (N = 25), Sham group (N = 25) and control group (N = 25), respectively. (**D**) depicts the startle responses of all participants (N = 75).

Startle responses indicate successful acquisition as both the main context effect (*F*(4,288) = 46.36, GG-ε = 0.818, *p* < 0.001, *ƞ*_*p*_^2^ = 0.392) and the interaction of Phase x Context (*F*(8,576) = 4.83, GG-ε = 0.839, *p* < 0.001, *ƞ*_*p*_^2^ = 0.063) were significant. Importantly, startle responses in CTX+ were potentiated compared to CTX− (*t*(74) = 2.62, *p* = 0.011) in the last trial of the second acquisition phase only (i.e., A5; see Fig. 2) which speaks for slow learning. Also notably, startle responses in both CTX+ and CTX− were in all acquisition phases higher compared to ITI (all *p*s ≤ 0.007) perhaps due to an increase in arousal.

#### Valence

**Figure 3:**
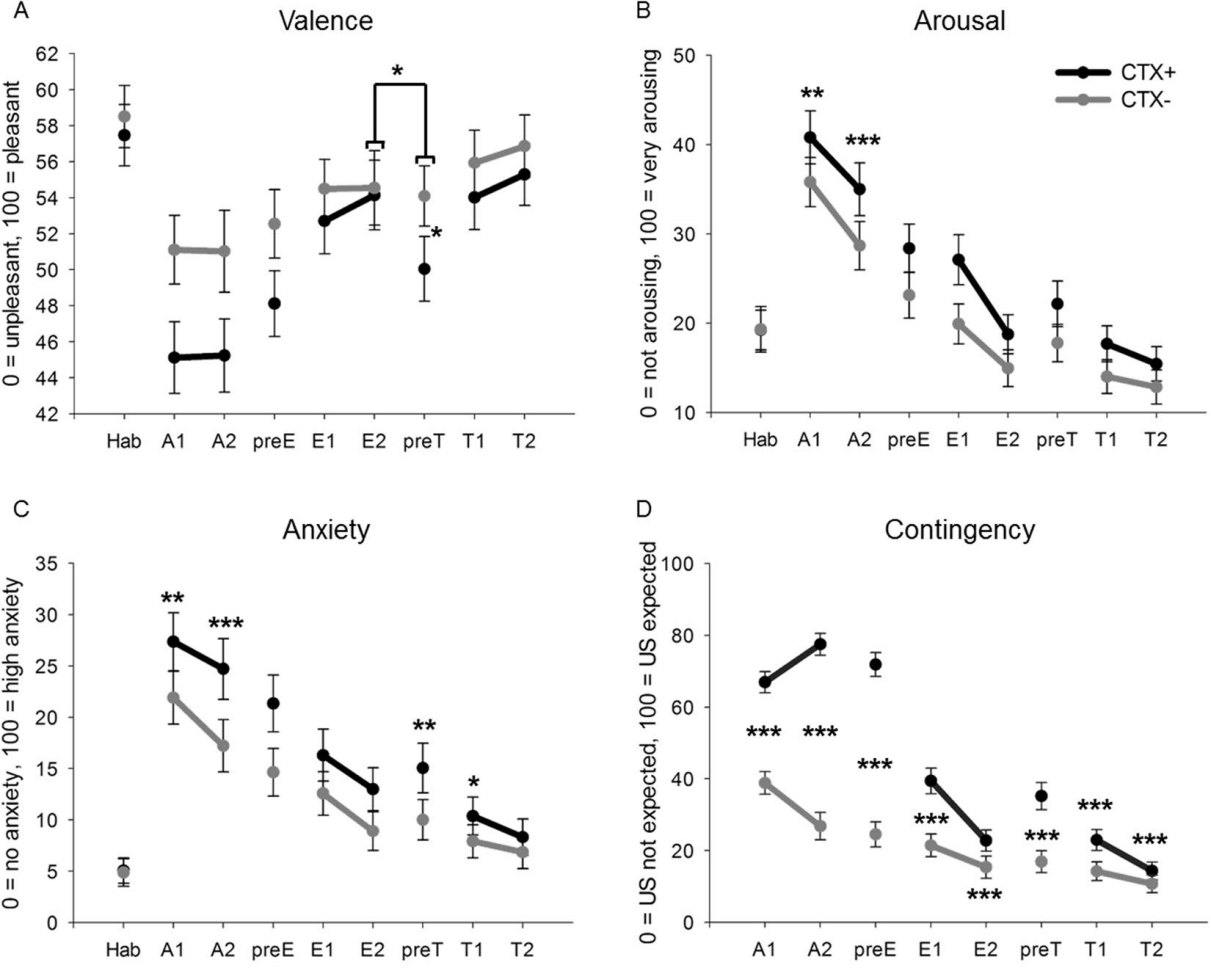
Ratings for context conditioning. Data for each rating were pooled across all three groups and are shown in one overall graph (N = 75). Circles (with standard errors) depict valence (**A**), arousal (**B**), anxiety (**C**), and contingency (**D**) ratings for anxiety context (CTX+) and safety context (CTX−). X-Axes show the time of the rating: After habituation (Hab), after Acquisition 1 (A1), and Acquisition 2 (A2) for Day 1, before (preE) and after Extinction 1 (E1) as well after Extinction 2 (E2) for Day 2, and for Day 3 before (preT) and after Test 1 (T1) and Test 2 (T2).

Valence was rated similar for both contexts after habituation (*t*(74) = 0.66, *p* = 0.509), but then during acquistion overall more negative for CTX+ as compared to CTX− (significant main effect context: *F*(1,72) = 8.11, *p* = 0.006, *ƞ*_*p*_^2^ = 0.101; non significant interaction Phase x Context: *F*(1,72) = 0.01, *p* = 0.940, *ƞ*_*p*_^2^ = 0.000) confirming a fast change in the valence of contexts (see Fig. 3A). A significant Phase x Group interaction (*F*(2,72) = 3.21, *p* = 0.046, *ƞ*_*p*_^2^ = 0.082) did not reach significance level in post-hoc *t*-tests (all *p*s ≤ 0.063).

#### Arousal

After habituation, both contexts elicited the same arousal in participants (*F*(1,72) = 0.00, *p* = 0.963, *ƞ*_*p*_^2^ = 0.000). A significant main effect of context (*F*(1,72) = 19.78, *p* < 0.001, *ƞ*_*p*_^2^ = 0.215) and a non-significant interaction Phase x Context (*F*(1,72) = 0.82, *p* = 0.368, *ƞ*_*p*_^2^ = 0.011) revealed that the arousal ratings were changed rapidly with higher arousal in CTX+ compared to CTX− (see Fig. 3B).

#### Anxiety

The anxiety ratings were similar for both contexts after habituation (*F*(1,72) = 0.09, *p* = 0.771, *ƞ*_*p*_^2^ = 0.001). Again a main effect of context (*F*(1,72) = 19.97, *p* < 0.001, *ƞ*_*p*_^2^ = 0.217) and a non-significant interaction of Phase x Context (*F*(1,72) = 1.26, *p* = 0.265, *ƞ*_*p*_^2^ = 0.017) revealed successful differential anxiety conditioning (see Fig. 3C).

#### Contingency

A significant main effect of context (*F*(1,72) = 68.93, *p* < 0.001, *ƞ*_*p*_^2^ = 0.489) and a significant interaction of Phase x Context (*F*(1,72) = 22.19, *p* < 0.001, *ƞ*_*p*_^2^ = 0.236) revealed successful acquisition with higher contingency ratings for CTX+ compared to CTX− for both A1 (*t*(74) = 5.83, *p* < 0.001) and A2 (*t*(74) = 8.65, *p* < 0.001) (Fig. 3D).

### Extinction of the conditioned contextual anxiety (Day 2)

On Day 2, participants were guided through both virtual offices again, but no US was delivered any more. For startle, arousal, and anxiety ratings during extinction, all effects involving the factor group were not significant (all *p*s ≥ 0.272) and therefore will not be reported.

#### Startle

The main effects of context (*F*(2,144) = 28.83, *p* < 0.001, *ƞ*_*p*_^2^ = 0.286) and the interaction Phase x Context (*F*(8,576) = 5.43, GG-ε = 0.819, *p* < 0.001, *ƞ*_*p*_^2^ = 0.070) revealed that CTX+ compared to CTX− triggered enhanced startle responses during E1 (*t*(74) = 2.51, *p* = 0.014), E2 (*t*(74) = 2.75, *p* = 0.007), and E3 (*t*(74) = 3.39, *p* = 0.001), but not during E4 and E5 (all *p*s ≥ 0.431; Fig. 2). Thus extinction of conditioned startle effects was successful.

#### Valence

In the pre-extinction ratings, the main effect of context did not reach significance (*F*(1,72) = 0.53, *p* = 0.053, *ƞ*_*p*_^2^ = 0.051). The interaction of Context x Group (*F*(2,72) = 3.64, *p* = 0.031, *ƞ*_*p*_^2^ = 0.092) showed more negative overall valence ratings in CTX+ compared to CTX− only in the control group (*t*(24) = 2.63, *p* = 0.015). A main effect of group revealed (*F*(2,72) = 4.21, *p* = 0.019, *ƞ*_*p*_^2^ = 0.105) more negative valence ratings in the VNS group compared to both the Sham (*t*(48) = 2.14, *p* = 0.037) and the control group (*t*(48) = 3.12, *p* = 0.003), which is in line with the more negatively rated valence of the tVNS stimulation itself. Effects of phase and context were not significant (all *p*s ≥ 0.366, Fig. 3A).

#### Arousal

Prior to extinction, a main effect of context (*F*(1,72) = 7.40, *p* = 0.008, *ƞ*_*p*_^2^ = 0.093) indicated still higher arousal in CTX+ compared to CTX−. Following up the significant interaction of Phase x Context (*F*(1,72) = 5.17, *p* = 0.026, *ƞ*_*p*_^2^ = 0.067) with post-hoc *t*-tests, we revealed higher arousal in CTX+ after E1 (*t*(74) = 4.42, *p* < 0.001) but also after E2 (*t*(74) = 2.91, *p* = 0.005). Therefore, we conclude that extinction learning happened but was not completely successful for arousal ratings (Fig. 3B).

#### Anxiety

A significant main effect of context in pre-extinction (*F*(1,72) = 10.34, *p* = 0.002, *ƞ*_*p*_^2^ = 0.126) and during extinction (*F*(1,72) = 11.73, *p* = 0.001, *ƞ*_*p*_^2^ = 0.140), but no significant interaction of Phase x Context (*p* = 0.783) indicate generally enhanced anxiety ratings in CTX+ compared to CTX−, demonstrating insufficient extinction learning (Fig. 3C).

#### Contingency

At pre-extinction, a main effect of context (*F*(1,72) = 81.76, *p* < 0.001, *ƞ*_*p*_^2^ = 0.532), but no group effects (all *p*s ≥ 0.148) indicate maintained associative learning in all groups. During extinction, a main effect of group (*F*(2,72) = 3.99, *p* = 0.023, *ƞ*_*p*_^2^ = 0.100) demonstrated higher contingency ratings of the VNS compared to the Sham group (*t*(48) = 3.04, *p* = 0.004). A main effect of context (*F*(1,72) = 28.16, *p* < 0.001, *ƞ*_*p*_^2^ = 0.281) and an interaction of Phase x Context (*F*(1,72) = 20.04, *p* < 0.001, *ƞ*_*p*_^2^ = 0.218) were due to higher contingency ratings in CTX+ than in CTX− for E1 (*t*(74) = 5.45, *p* < 0.001), and still for E2 (*t*(74) = 4.24, *p* < 0.001); thus extinction learning occurred but was not complete (Fig. 3D).

### Reinstatement of conditioned contextual anxiety (late Day 2 vs. early Day 3)

Reinstatement effects induced by delivering three unannounced US at the beginning of Day 3 are indicated either by a general increase in responses from end of extinction to the first test trial (general reinstatement; significant phase effect) or by a resurge of a CTX+ versus CTX− difference (differential reinstatement; significant phase x context interaction). Reinstatement effects were only tested for variables with successful extinction, i.e. startle responses and valence rating. All effects involving the factor group were not significant and therefore will not be reported.

#### Startle

The ANOVA revealed main effects of phase (*F*(1,72) = 56.89, *p* = 0.001, *ƞ*_*p*_^2^ = 0.440) and context (*F*(2,144) = 19.21, *p* = 0.001, *ƞ*_*p*_^2^ = 0.211) as well as an interaction of Phase x Context (*F*(2,144) = 13.48, *p* < 0.001, *ƞ*_*p*_^2^ = 0.158; Fig. 2). Post-hoc *t*-tests for the significant interaction indicated return of anxiety for all conditions. In fact, startle responses were significantly potentiated during the first test trials as compared to the last extinction trials for CTX+ (*t*(74) = 7.78, *p* < 0.001), CTX− (*t*(74) = 6.17, *p* < 0.001) and ITI (*t*(74) = 2.60, *p* = 0.011), and at the first test trials we observed no difference between CTX+ and CTX− (*t*(74) = 1.10, *p* = 0.247). Importantly, startle responses to both CTX+ (*t*(74) = 5.64, *p* < 0.001) and CTX− (*t*(74) = 5.26, *p* < 0.001) were significantly increased compared to ITI, while this differences were not significant at the last extinction trials, thus we observed a general reinstatement.

#### Valence

A main effect of phase (*F*(1,72) = 4.24, *p* = 0.043, *ƞ*_*p*_^2^ = 0.056), but not context (*F*(1,72) = 2.15, *p* = 0.147, *ƞ*_*p*_^2^ = 0.029), and a significant interaction of Phase x Context (*F*(1,72) = 4.75, *p* = 0.033, *ƞ*_*p*_^2^ = 0.062, Fig. 3A) were followed up with post-hoc *t*-tests showing that CTX+ and CTX− were rated similarly at the last extinction phase (*t*(74) = 0.27, *p* = 0.788), whereas after reinstatemet CTX+ was rated significantly more negative than CTX− (*t*(74) = 2.08, *p* = 0.041). Therefore, valence ratings reveal a differential return of conditioned valence after reinstatment.

### Re-extinction (Day 3)

Re-extinction is determined by analysis of startle responses on Day 3 and comparison of the ratings after the first and the second test phase. Neither main effects of group nor interactions with group could be found for startle response, arousal and anxiety ratings (all *p*s ≥ 0.080).

#### Startle

A main effect of context (*F*(2,144) = 42.56, GG-ε = 0.733, *p* < 0.001, *ƞ*_*p*_^2^ = 0.372) and an interaction of Phase x Context (*F*(8,576) = 4.54, GG-ε = 0.846, *p* < 0.001, *ƞ*_*p*_^2^ = 0.059, Fig. 2) was due to potentiated startle in CTX+ compared to ITI in T1 (*t*(74) = 6.94, *p* < 0.001), T2 (*t*(74) = 2.30, *p* = 0.024), T3 (*t*(74) = 6.33, *p* < 0.001), and T4 (*t*(74) = 3.89, *p* < 0.001), but not in T5 (*t*(74) = 1.06, *p* = 0.291), and due to potentiated startle for CTX− compared to ITI in T1 (*t*(74) = 5.54, *p* < 0.001), T3 (*t*(74) = 5.03, *p* < 0.001), T4 (*t*(74) = 2.48, *p* = 0.015), and T5 (*t*(74) = 2.04, *p* = 0.045), but not in T2 (*p* = 0.136). No differences between CTX+ and CTX− in T1, T2, T3 and T5 (all *p*s ≥ 0.150), but in T4 (*t*(74) = 2.36, *p* = 0.021) were found. Overall, these results indicate re-extinction of the induced general reinstatement effect.

#### Valence

Since we revealed neither an effect of context nor an interaction of Phase x Context (all *p*s ≥ 0.185, Fig. 3A) we conclude that re-extinction of the induced differential reinstatement effect was successful. An interaction of Phase x Group (*F*(2,72) = 4.88, *p* = 0.010, *ƞ*_*p*_^2^ = 0.119) indicates that in the Sham group valence increased significantly from T1 to T2.

#### Arousal

The main effect of context (*F*(1,72) = 9.91, *p* = 0.002, *ƞ*_*p*_^2^ = 0.121) and the absent interaction of Phase x Context (*F*(1,72) = 0.78, *p* = 0.380, *ƞ*_*p*_^2^ = 0.011; Fig. 3B) indicate insufficient re-extinction with generally higher arousal ratings in CTX+ compared to CTX−.

#### Anxiety

A non-significant effect of context (*F*(1,72) = 3.90, *p* = 0.052, *ƞ*_*p*_^2^ = 0.051) and a non-significant interaction of Phase x Context (*F*(1,72) = 2.02, *p* = 0.159, *ƞ*_*p*_^2^ = 0.027; Fig. 3C) confirm that re-extinction was successful.

#### Contingency

Main effects of group (*F*(2,72) = 4.56, *p* = 0.014, *ƞ*_*p*_^2^ = 0.112), context (*F*(1,72) = 18.91, *p* < 0.001, *ƞ*_*p*_^2^ = 0.208), and an interaction of Phase x Context (*F*(1,72) = 12.11, *p* = 0.001, *ƞ*_*p*_^2^ = 0.144; Fig. 3D) were revealed. Post-hoc *t*-tests returned higher contingency ratings for CTX+ compared to CTX− after preT (*t*(74) = 4.76, *p* < 0.001), after T1 (*t*(74) = 4.55, *p* < 0.001), and after T2 (*t*(74) = 2.87, *p* = 0.005). Following up the main effect for group, *t*-tests revealed lower contingency ratings in the Sham group compared to the VNS group (*t*(48) = 3.42, *p* = 0.002) and compared to controls (*t*(48) = 2.13, *p* = 0.040), but no difference between the VNS group and controls (*t*(48) = 1.05, *p* = 0.301). Thus, we observed some re-extinction which however was not completely successful, and we observed some stimulation effects which however were unrelated to conditioning.

## Discussion

The first aim of this study was to experimentally demonstrate return of anxiety by means of reinstatement with a three days virtual reality context conditioning paradigm. Examining acquisition, extinction and reinstatement on different days is crucial since this ensures memory consolidation between learning phases. Our second goal was to translate animal findings on the positive effects of vagus nerve stimulation (VNS) on extinction and return of anxiety^34^ to humans using transcutaneous vagus nerve stimulation (tVNS). Demonstrating such effects in humans would open new options for the treatment of anxiety disorders.

Regarding the first goal, this study revealed successful acquisition of contextual anxiety, i.e. stronger anxiety response in the anxiety (CTX+) compared to the safety (CTX−) context, for both physiological (startle responses) and verbal (ratings of valence, arousal, anxiety, and contingency) measures. These conditioning effects were still apparent before extinction on Day 2, substantiating consolidated anxiety memory traces. The subsequent extinction phase proved successful for startle responses and valence ratings, however not for arousal, anxiety and contingency ratings. Such dissociations in extinction learning between different anxiety measures were also found in several other studies^13,43^. Haaker *et al*.^44^ discussed this issue in a review. Apparently, the CS-US association during acquisition requires less trials than the formation of the CS-noUS association during extinction. On evolutionary perspective, fight or flight in response to an ambiguous stimulus seems to be the better strategy for survival. In order to experimentally strengthen the extinction memory particularly for ratings more extinction trials might be necessary.

Reinstatement on Day 3 was found for both valence ratings and startle responses, however, with diverging effects. On the one hand, we observed differential reinstatement^44^ for valence ratings, thus enhanced anxiety responses in CTX+ only. On the other hand, we revealed generalized reinstatement for startle responses, thus potentiated startle responses in both CTX+ and CTX− compared to ITI. These results corroborate Glotzbach-Schoon *et al*.^22^ who used a similar VR paradigm and Haaker *et al*.^45^ who used pictures of a context in a cue in context paradigm and also reported generalized reinstatement regarding fear potentiated startle and differential reinstatement regarding anxiety ratings. Reinstatement of conditioned anxiety supports the idea that anxiety memories are not erased during extinction learning^17^. Notably, remembered aversive life events have an important role from an evolutionary perspective^46^. Consistently, Hamm and Weike^47^ as well as LeDoux and Pine^48^ suggest a two-system framework relying on a cognitive level of fear, which can be measured by ratings, and on a defensive survival circuit, which evokes defensive behaviour as well as physiological responses. This model explains dissociative results in cognitive anxiety representing mental states, and physiological as well as behavioural responses regarding defensive behaviour, physiological adjustment and brain responses^48^.

The present findings are highly comparable to those reported by Glotzbach-Schoon *et al*.^22^, although we had to vary several experimental parameters for the present experiment, e.g. the length and number of trials, the number of US for reinstatement and the used VR technology (Powerwall instead of head-mounted display). Recently, Richter^49^ emphasized the importance of systematic heterogenization of experimental protocols in order to demonstrate the validity of study designs. Therefore, this study allows the substantiated conclusion that the conducted three days context conditioning paradigm is reliable and valid to examine new ways to modulate return of anxiety induced by reinstatement. VR proved to be an elegant and ergonomic tool investigating contextual anxiety within a naturalistic and highly controlled experimental setup^12,22,50,51^.

Our second goal was the translation of findings in animals that VNS accelerates and stabilizes extinction memory^34^. From a mechanistic point of view, VNS should increase norepinephrine (NE) release in brain areas associated with extinction learning like medial prefrontal cortex (mPFC) and basolateral Amygdala (BLA)^20^. Such NE release may facilitate neural plasticity resulting in stronger infra-limbic (IL)-BLA pathways leading consequently to stronger extinction memory traces^35^. Interestingly, comparable results in rats were not only found for extinction of conditioned fear or anxiety, but also for extinction of drug seeking behaviour^52^.

The present study in humans found no reliable effects of tVNS on extinction or on reinstatement of contextual anxiety. This lack of tVNS effects holds true for both physiological and verbal measure of anxiety and for measures with complete or incomplete extinction. Indeed, we observed successful extinction as reflected in comparable responses to the anxiety and the safety context for startle responses and valence ratings, but not for ratings of arousal, anxiety and contingency. However, as we found no tVNS effects for all examined parameters we feel confident to argue that tVNS - as realized here - was not effective in improving extinction or preventing return of anxiety.

The most obvious reason for the lack of translational success might by the stimulation methodology, VNS versus tVNS. However, as direct stimulation via the neck portion of the vagus nerve requires surgery in animals as well as in humans, human studies with VNS can only be performed with patients wearing an implanted stimulator for medical reasons^53^, e.g. epilepsy. Thus, we consider human studies employing tVNS to improve fear extinction a promising and clinically highly interesting approach, although the electrical stimulation of the auricular branch of the vagus nerve might be rather weak. This study is a first important step which we are convinced will lead to improved stimulation parameters and experimental designs. Information about the most effective stimulation parameters are still very limited. According to the functional magnetic resonance imaging (fMRI) study of Frangos *et al*.^41^, activation of brain areas like nucleus tractus solitarius (NTS) and amygdala needs at least seven minutes of constant cymba conchae stimulation. Even though we used 20 min interval stimulation prior to extinction and during extinction trials, no effects were revealed. Possibly, our transcutaneous stimulation interval might have been still too short for being effective, since some studies reported physiological effects of tVNS when applied few hours per day for several weeks^54^. In addition to stimulation duration, stimulation timing seems crucial. Animal studies revealed that precise timing of VNS with a sensory event or motor response induces cortical plasticity^55^. Therefore, we exactly paired the stimulation during extinction with the participants’ stay in the offices, and suggest that future studies should continue with this approach. Importantly, future human studies have to incorporate reliable indicators of vagal activity. Our manipulation check for tVNS stimulation, heart rate changes, did not return significant effects (see supplementary material). More reliable indicators for vagal activity could be heart rate variability^56^ or measure of pupil dilation^57^ and/or analgesic effect of tVNS^58^. Further human investigations with a valid manipulation check are indispensable to draw conclusions about tVNS effects on fear extinction. Finally, the conditioning protocol might by important. Most animal conditioning experiments and similarly Peña *et al*.^34^ used one stimulus (e.g. tone) or context (e.g. cage) only which becomes associated with the US during acquisition. In contrast, most human studies and similarly this study employed differential conditioning paradigms which in our case was realized with two contexts, an anxiety and a safety context. This difference may be crucial, as a meta-analysis by Lissek *et al*.^59^ suggests. Consequently, applying a single stimulus or context conditioning paradigm in humans might help in translating animal conditioning research on VNS to humans.

Human studies investigating effects of VNS or tVNS on extinction learning are still sparse. George *et al*.^60^ examined anxiety disorder patients who were resistant to conventional treatment and received an implanted vagus nerve stimulator. They found improvement of anxiety symptoms in one third of the patients suffering from obsessive compulsive disorder (OCD), panic disorder (PD), or post-traumatic stress disorder (PTSD). Recently, Safi *et al*.^39^ supported the suggestion that tVNS could be an alternative to invasive (i)VNS based on their findings that myelinated afferent A beta fibers, which presumably mediate the stimulation effect in iVNS, are present at relative high numbers also in the human ABVN. In accordance with this, Burger *et al*.^42^, who investigated extinction learning in a fear conditioning paradigm in healthy participants using tVNS, found lower overall online contingency ratings during extinction in the tVNS compared to a sham stimulated group. The extinction learning curves differed mainly in the first trials of extinction. These results could serve as a first indicator that tVNS might have an effect on cognitive extinction learning in humans. However, in our study the tVNS compared to the Sham group revealed a trend for higher contingency ratings. These discrepancies may be explained by methodological differences like online vs. offline ratings, fear vs. anxiety conditioning, or different stimulation intensities^29^. Only two contingency ratings during extinction in our paradigm could be too few to assess the learning curve during initial extinction trials. Moreover, Burger *et al*.^42^ used the same intensity (i.e., 0.5 mA) for all participants, whereas we adjusted the intensity individually leading to a higher mean intensity (*M* = 1.2, *SD* = 1.1). Our explorative analyses revealed that a higher stimulation intensity in the VNS group was associated with higher startle amplitudes in the safety context after reinstatement indicating impaired safety learning during extinction (see supplementary material). Thus, the high stimulation realized here may have caused distraction from the extinction learning process.

Unlike previous studies, which mainly compared a tVNS with a sham stimulation group, we additionally included a second control group without any stimulation and therefore are able to discuss unspecific effects of stimulation. These analyses revealed differences between the unstimulated group and the stimulated groups in the valence ratings for CTX+ compared to CTX− on Day 2 before extinction actually started. Interestingly, the unstimulated group rated the CTX+ as more negatively valenced than the CTX− while this difference was not significant in both stimulated groups. Firstly, we speculate that the 20 min. interval stimulation influenced valence ratings, made them more similar. Secondly, we think that a context effect played a crucial role since the stimulation itself constituted a context, thus only the control group remained in the exactly same context. Future studies should consider and control such context effects^61^.

Recently, several lines of research investigated the improvement of extinction learning and exposure therapy. Besides pharmacological approaches^62–64^ or stress induction^65^, behavioural approaches are discussed which as emphasized by Pittig *et al*.^66^ might be procedural strategies during extinction, e.g., multiple context exposure^67^, or flanking strategies applied prior and post extinction, e.g., induction of positive affect which might positively influence safety learning^68^. Considering the latter distinction, VNS seems to serve as both, a flanking strategy, as it might be used in preparation for extinction learning, and a procedural strategy, as it is most effective when the stimulation is paired with the extinction stimulus^34^. Therefore, tVNS is a promising method, and additional research on its effects on extinction learning is needed.

In sum, extinction is the experimental analogue of exposure-based therapy for anxiety disorders^69^. Therefore, well established experimental paradigms are required, as the three days virtual reality context conditioning paradigm described here. Subsequent investigations on the enhancement of extinction learning and the prevention of return of anxiety by means of tVNS might help to further improve exposure therapy.

## Material and Methods

### Sample

In total, 93 participants were recruited to take part in the experiment. Exclusion criteria were alcohol abuse, intake of centrally affective drugs, neurological or psychiatric disorders, eye-sight or hearing problems and pregnancy. In addition, participants had to be naïve in respect to tVNS and did not know the virtual environment we used. Data of 75 participants (41 females; age: *M* = 24.61 years, *SD* = 3.23) could be included into analyses. Eighteen participants had to be excluded due to either technical problems in any part of the experiment (*N* = 9), non-responder (*N* = 6, see Data Reduction for criteria), or cancellation of the experiment early (*N* = 3). Prior to the start of the experiment, participants were assigned to either real stimulation (tVNS), sham stimulation (Sham), or control (control) group. Groups did not significantly differ in gender, age and trait anxiety (Table 2). Additionally, after acquisition participants were asked to indicate in which context they received electric stimuli. Participants that reported the correct context were labelled as aware participants, numbers did not differ between groups (Table 2). Analyses without unaware participants resulted in the same effects as reported above. Therefore, we conclude that awareness did not influence our results. All participants gave their written informed consent. For completing the full experiment, participants obtained 36 € for compensation. The experiment was performed in accordance with relevant guidelines and regulations. The study was approved by the Ethics Committee of the Medical Faculty of the University of Würzburg.

**Table 2:** Sample characteristics separated for the three groups: Verum stimulated participants (VNS), sham stimulated participants (Sham), and participants assigned to the control group (Control), who did not perceive any stimulation.

	VNS	Sham	Control	statistics
N (females)	25 (14)	25 (14)	25 (13)	*Χ* ^2^(2) = 0.948
Age (SD)	24.9 (3.6)	24.3 (2.7)	24.6 (3.6)	*p* = 0.831
Aware	19	21	17	*p* = 0.405
Stimulation intensity [mA] (SD)	1.2 (1.1)	1.0 (0.6)	—	*p* = 0.338
Stimulation rating (SD)	6.9 (0.7)	6.8 (1.6)	—	*p* = 0.739
US intensity [mA] (SD)	2.0 (1.6)	2.7 (1.7)	2.3 (1.6)	*p* = 0.393
US rating	5.8 (0.9)	5.5 (1.0)	5.8 (1.0)	*p* = 0.449

### Stimuli and Apparatus

#### Virtual reality

The equipment of virtual environment was already used and published in several other studies^51^. Shortly, the VR environment was created with Source Engine (Velve Corporation, Bellevue, USA) and contained two distinguishable offices with similar furniture. The offices were connected by a corridor serving as inter-trial interval (ITI). Neutral wall pictures were taken from the International Affective Picture System (1121, 5390, 5395, 7160, 7247, 7248, 7249, 7547, 7820, 7830)^70^. Participants were sitting 1.5 m in front of the Powerwall, a screen of 2 m in height and 3.22 m in length, to which the virtual environment was projected in nearly real size. The advantage of this technology is that participants can still see their own body while they are in the virtual environment which increases the presence feeling. Experimental control was established using VR-software CyberSession (CS-Research 5.6, VTplus GmbH, Würzburg, Germany; see www.cybersession.info for detailed information).

#### Unconditioned stimulus (US)

We used an electric stimulus as US with a frequency of 50 Hz and a duration of 200 ms generated by a constant current stimulator (Digitimer DS7A, Digitimer LTD., Welwyn Garden City, UK). The US was applied with a surface electrode fixated on the inner side of the right forearm. In order to create a mildly painful electric stimulus for each participant, the individual pain threshold was determined according to the procedure in Andreatta *et al*.^71^. For the experiment, the current intensity was increased by 30%^72^. The determined mean stimulation intensity for the US was 2.34 mA (*SD* = 1.65) and the rating was 5.72 (*SD* = 0.97). Both did not differ between groups (all *p*s ≥ 0.393; see Table 2).

#### Vagus nerve stimulation

Electrical VNS was applied on Day 2 using NEMOS, a transcutaneous vagus nerve stimulator developed by cerbomed GmbH (Erlangen, Germany). For the tVNS group, the device was applied at the cymba concha, for the Sham group, the stimulation was applied at the helix of the outer ear (see supplementary material). For the control group, the tVNS device was attached to the cymba concha, but never switched on. The stimulation intensity was determined for each participant assigned to the verum or Sham group individually. In order to evoke the optimal stimulation, a tingling sensation was required, but by no means pain^58^. The stimulation consisted of rectangular pulses of 250 µS at 25 Hz. For tingling threshold, 10 s of stimulation intervals were administered with increasing intensities in steps of 0.1 mA. Participants had to rate the feeling of each stimulation on an 11-point Likert scale from 0 (no sensation) to 3 (slight tingling) to 6 (strong tingling) to 10 (painful). When participants rated the intensity above 7, a decreasing series of stimulation intensities in steps of 0.1 mA followed until participants rated the stimulation below 7. This procedure was then repeated one more time. In the end, the mean stimulation intensity of the two increasing and the two decreasing series was calculated, that was rated as 7. Finally, a 10 s stimulation interval with the determined intensity should be rated as 6 or 7. If this was the case, the determined stimulation intensity was used throughout the whole experiment. If the rating was above or below 6 or 7, the intensity was adapted until the required rating was made. Stimulation intensity and stimulation rating did not differ between stimulated groups (all *p*s ≥ 0.338; see Table 2). After the stimulation threshold was determined VNS and Sham group participants got a 20 min. interval stimulation (30 s on and 30 s off phases) since Frangos *et al*.^41^ found neurological effects of the stimulation with temporal latency. The stimulation during extinction was synchronized with the stay of the participants in the virtual offices.

### Measures

#### Questionnaires

Prior to the experiment, participants completed the following questionnaires: A demographic questionnaire containing age, gender, education, profession and handedness. The German version of the Anxiety Sensitivity Index ASI^73^ measures participant’s anxiety and anxious experiences. Participant’s general and current anxiety was assessed by the German version of the State-Trait Anxiety Inventory^74^. In order to record participant’s emotional state before and after the experimental procedure, the German version of the Positive Affect Negative Affect Schedule was completed^75^ (see supplementary table). Moreover, one questionnaire about the tVNS was assessed in the end of Day 2. Here, participants had to indicate whether they thought, the stimulation worked (0 = stimulation did not work; 10 = stimulation worked very well), valence of the stimulation (0 = unpleasant; 10 = pleasant) as well as their subjective conviction of the functionality (0 = not convinced; 10 = very convinced).

#### Ratings

Ratings of valence, arousal, anxiety and contingency were assessed for each context using a 100-point Likert scale presented on the Powerwall. In addition to each scale, a picture of the referring context was depicted. Participants reported their ratings verbally. The experimenter wrote the ratings down on a protocol sheet. The valence scale ranged from 0 (very unpleasant), 50 (neutral) to 100 (very pleasant), the arousal and anxiety scales from 0 (not arousing/not anxious) to 100 (very arousing/very anxious) and the contingency scale from 0 (surely no US) to 100 (surely US).

#### Startle

The startle probes were white noises presented for 50 ms via headphones binaurally with 103 dB. In order to measure the startle response, electromyographic activity (EMG) was recorded from M. orbicularis oculi. Electrodes were positioned below the left eye, one electrode was placed centrally below the pupil and the second electrode about 1 cm aside^76^. The reference electrode was placed on the forehead and the ground electrode on the left mastoid. The impedances of all electrodes were kept below 10 kΩ. Physiological data were continuously recorded with the Vision Recorder software (Brain Products Inc., Munich, Germany). The EMG sampling rate was at 1000 Hz, an online Notch filter was applied at 50 Hz.

### Experimental procedure

The experimental procedure was based on the study by Glotzbach-Schoon *et al*.^22^. The following modifications were introduced in order to adapt the paradigm for manipulations required for the vagus nerve stimulation in 30 s intervals: The trials were shorter (30 s instead of 85 s per context), more trials per context were performed (5 instead of 3 per phase). Additionally, we used the Powerwall instead of a head-mounted display and we administered three instead of one US for reinstatement.

In detail, the experiment was performed on three consecutive days, separated by 24 h (Fig. 1). On Day 1, participants completed the questionnaires and the electrodes for physiological recordings were applied. Afterwards, participant’s individual pain threshold was determined as described earlier. During the initial habituation phase (Hab), participants saw the corridor of the virtual environment on the Powerwall and were instructed to freely walk into both offices by means of a joystick. They had 2 min for inspecting each office. Subsequently, participants rated the context according to valence, arousal and anxiety. Next, seven startle probes were presented in intervals between 9 s and 17 s in order to minimize initial startle reactivity. During the two acquisition phases, participants were passively guided through the virtual environment on one of two alternating pre-recorded paths per context (clockwise and counter-clockwise walking). One trial consisted of a path that lasted about 55 s, starting in the corridor (ca. 14 s), walking around in an office for 30 s, and ending in the corridor again (ca. 8 s). Both Acquisition 1 (A1) and Acquisition 2 (A2) consisted of five trials for each office. During each trial, startle probes could be delivered in the corridor (0 or 1 startle probes, randomly before the entrance or after the exit of an office) as well as in the offices (randomly, at least 6 s after entering an office, 1 or 2 startle probes per trial). In total, 12 startle probes were presented in the corridor and 12 in each office. The minimum interval between startle probes or electric stimuli was 9 s. Importantly, in one office (anxiety context or CTX+), but not in the other office (safety context or CTX−), participants could unpredictably receive 0 to 2 painful USs per trial. Altogether, 12 electric stimuli were applied (i.e., 6 each acquisition phase). Participants were instructed that it is possible to predict the electrical stimuli^77^ in order to focus participants’ attention on the context-US association. After A1 and A2 ratings of valence, arousal, anxiety and contingency were assessed for CTX+ and CTX−. Contexts as well as the order of entering the rooms were counterbalanced across participants.

Day 2 started with the completion of PANAS and STAI state questionnaires. Afterwards, the vagus nerve stimulator was inserted. In both tVNS and Sham groups the stimulation threshold determination was performed (see Stimulus Material). Prior to the start of the experiment, both groups were stimulated with the determined intensity for 20 min. In line with the stimulation rhythm of the tVNS device for epilepsy patients (cerbomed, Erlangen, Germany), the 20 min stimulation consisted of intervals of 30 s stimulation followed by 30 s break. In the meantime, the electrodes for both the EMG and the US were attached like on Day 1. The control group perceived no stimulation while all electrodes were attached. After these preparations and the 20 min interval stimulation two extinction phases (E1 and E2) were conducted. These phases worked exactly like the acquisition phases on Day 1 except that no US was delivered. Again, altogether 12 startle probes were delivered in the corridor (0 to 1 per trial), in CTX+ (1 to 2 per trial) and in CTX− (1 to 2 per trial), respectively. Vagus nerve or Sham stimulation synchronized with the entering of an office in order to stimulate the participants specifically throughout the context duration. Valence, arousal, anxiety and contingency were rated in the beginning (preE) and after E1 and E2.

On Day 3, all electrodes were applied and while a black screen was shown on the Powerwall three unannounced USs were presented for reinstatement of the conditioned anxiety^78^. Afterwards, participants underwent two test phases (T1 and T2), which worked exactly as the two extinction phases of Day 2 and no further US was administered. Ratings were assessed before start of the test phase (preT) and after T1 and T2.

### Data reduction and analysis

#### Startle response

The EMG data were processed with Vision Analyzer 2.1 software (Brain Products Inc., Munich, Germany). A low cut-off filter of 28 Hz and a high cut-off filter of 500 Hz were applied to the offline data. Afterwards, the signal was rectified, smoothed with a moving average window of 50 ms and baseline corrected from −50 ms to the startle probe onset^79^. Startle peaks were detected in a time window between 20 and 200 ms after startle probe onset. Artefacts were manually scored and defined as baseline shifts higher than 5 µV. Non-responders were defined as those participants with a mean startle magnitude below 5 µV and excluded from analyses. In order to control for general differences in startle responses between participants, raw data were transformed into *z*-scores within subjects and subsequently in T-scores. Missing startle responses were interpolated within subject and condition. Afterwards, T-scores of 2 consecutive trials were averaged and reported as phase. Consequently, each day resulted in 5 phases, which is referred to as A1-A5 (acquisition), E1-E5 (extinction), and T1-T5 (test).

### Statistical analysis

Repeated measures ANOVAs were calculated for acquisition, extinction and test separately including the between-subjects factor group (VNS, Sham, control). For startle response, we calculated three 3 × 5 ANOVAs for each part separately with the within-subject factors context (CTX+, CTX−, ITI) and phase (A1-A5, E1-E5, and T1-T5, respectively) and a 3 (context: CTX+, CTX−, ITI) × 2 (phase: last phase of extinction, first phase of test) ANOVA for reinstatement. The ratings were analysed by 2 × 2 ANOVAs with the within-subject factors context (CTX+, CTX−) and phase (after the first and after the second acquisition, extinction and test phase/re-extinction, respectively). Reinstatement of conditioned anxiety was tested by a separate 2 (context: CTX+, CTX−) × 2 (phase: E5, T1) ANOVA.

The state questionnaires PANAS positive affect and negative affect as well as STAI state were analysed by an ANOVA with the within-subject factor phase (acquisition, extinction, test) and the between-subjects factor group (VNS, Sham, control). ASI and STAI trait were compared between groups with one-way ANOVAs.

Post-hoc *t*-tests were calculated for significant main effects and interactions. In case of violencing sphericity, Greenhouse-Geisser corrections were applied and Greenhouse-Geisser Epsilon (GG-ε) reported. The α-niveau was set at 0.05 for all statistical tests.

## Electronic supplementary material

Supplementary Material
