# Supplementary material for: Reinstatement of contextual conditioned anxiety in virtual reality and the effects of transcutaneous vagus nerve stimulation in humans
Source: Sci Rep. 2017 Dec 20;7:17886. doi: 10.1038/s41598-017-18183-3 (PMC5738426; doi:10.1038/s41598-017-18183-3)
Supplement: Supplementary file 1 — Supplementary Material [file 41598_2017_18183_MOESM1_ESM.doc]

# Reinstatement of contextual conditioned anxiety in virtual reality and the effects of transcutaneous vagus nerve stimulation in humans

Hannah Genheimer^1*^, Marta Andreatta^1^, Esther Asan^2^, Paul Pauli^1,3^

*Corresponding author

^1^Department of Psychology (Biological Psychology, Clinical Psychology, and Psychotherapy), University of Würzburg, Germany

^2^Institute of Anatomy and Cell Biology, University of Würzburg, Germany

^3^Center of Mental Health, Medical Faculty, University of Würzburg, Germany

# Supplementary Material


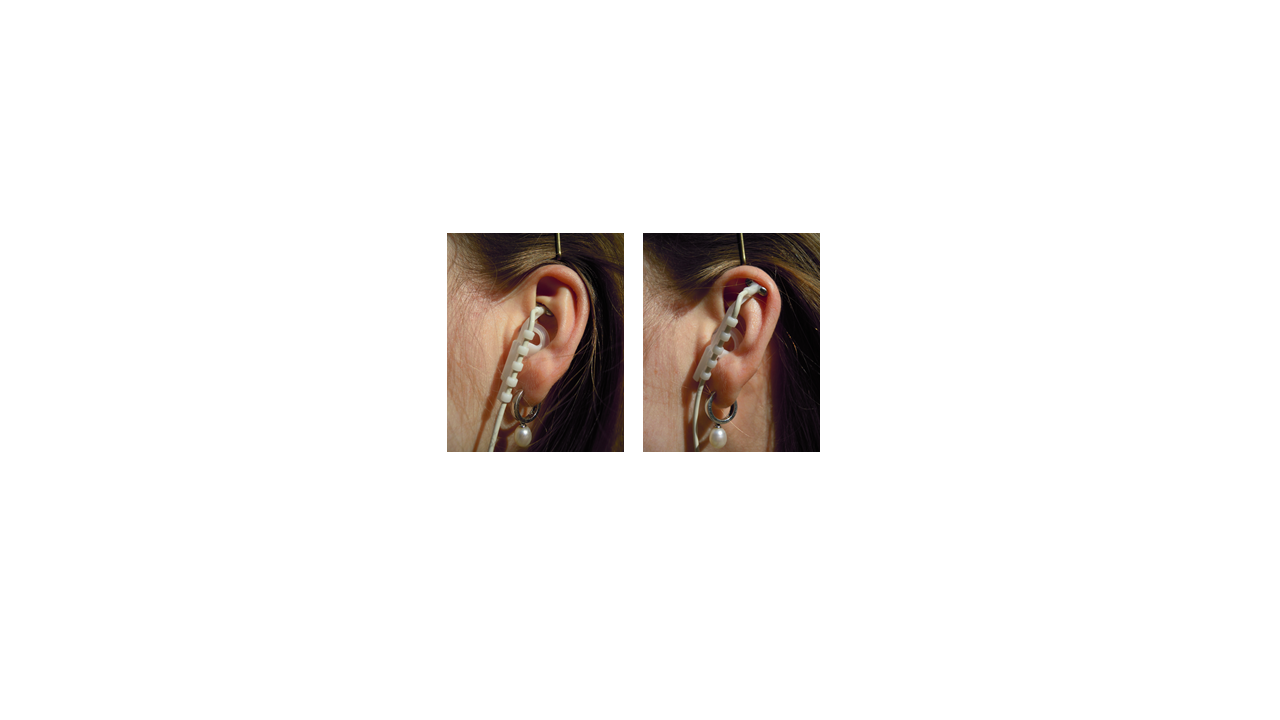


Suppl. Figure S1: Stimulation sites. The stimulator was applied to the left cymba concha in the VNS group (left) and to the left helix in the Sham group (right). In the control group, the stimulator was applied as shown on the left panel, but the device was never turned on.

## Subjective effects

Several clinical studies with patients wearing an implanted vagus nerve stimulator reported positive affect and anti-depressant effects due to stimulation. We used the anxiety sensitivity index (ASI)[^68^](#_ENREF_68), the positive affect, negative affect scale (PANAS)[^70^](#_ENREF_70) and STAI state and trait[^69^](#_ENREF_69) to assess participants’ feelings.

Regarding ASI, the one-way ANOVA revealed differing anxiety sensitivity between groups (*F*(2,85) = 3.96, *p* = .023, *ƞ_p_^2^* = .099) indicating that the Sham group had a lower anxiety sensitivity index compared to both VNS (*t*(48) = 2.75, *p* = .009) and to controls (*t*(48) = 2.08, *p* = .043) (Suppl. Table). No differences in PANAS and STAI state on acquisition, extinction and test day were found among groups. However, the ANOVA revealed a main effect of phase for PANAS positive affect (*F*(2,144) = 19.18, *p* < .001, *ƞ_p_^2^* = .210) and PANAS negative affect (*F*(2,144) = 4.01, GG-ε = .757, *p* = .031, *ƞ_p_^2^* = .053). Post-hoc *t*-tests indicated higher positive affect on acquisition day compared to extinction day (*t*(74) = 5.51, *p* < .001) and to test day (*t*(74) = 5.26, *p* < .001). Furthermore, the negative affect ratings were also higher on acquisition day compared to test day (*t*(74) = 2.76, *p* = .007). Neither temporal effects of STAI state questionnaire were found (all *ps* ≥ .089) nor group effects of STAI trait questionnaire (*F*(2,72) = 1.91, *p* = .156, *ƞ_p_^2^* = .050) (see suppl. table).

In sum, we could not find group differences in state questionnaires PANAS and STAI throughout the experimental procedure which might be due to the relatively short stimulation period[^53^](#_ENREF_53).

Suppl. Table 1: Anxiety Sensitivity Index, PANAS positive affect, negative affect and STAI state and trait and the comparison between groups (VNS, Sham, control).

|  | **VNS** | **Sham** | **Control** | **statistics** |
| --- | --- | --- | --- | --- |
| ASI (SD) | 18.4 (9.1) | 12.3 (6.5) | 16.6 (8.0) | *p* = .023* |
| STAI trait (SD) | 39.5 (9.4) | 34.88 (8.8) | 39.1 (9.5) | *p* = .156 |
| Positive affect day 1 (SD) | 30.5 (5.7) | 32.4 (5.6) | 32.1 (5.7) | *p* = .441 |
| Positive affect day 2 (SD) | 26.5 (6.6) | 30.4 (5.6) | 29.0 (8.1) | *p* = .133 |
| Positive affect day 3 (SD) | 25.5 (7.4) | 29.8 (7.1) | 29.0 (6.9) | *p* = .086 |
| Negative affect day 1 (SD) | 12.8 (3.3) | 11.8 (2.4) | 14.0 (4.9) | *p* = .107 |
| Negative affect day 2 (SD) | 12.2 (3.4) | 11.5 (3.1) | 12.7 (4.3) | *p* = .484 |
| Negative affect day 3 (SD) | 11.8 (2.6) | 11.3 (2.1) | 11.9 (2.6) | *p* = .683 |
| STAI state day 1 (SD) | 35.0 (7.3) | 33.4 (5.9) | 36.6 (8.1) | *p* = .291 |
| STAI state day 2 (SD) | 36.3 (8.0) | 34.3 (7.5) | 37.4 (11.6) | *p* = .475 |
| STAI state day 3 (SD) | 35.5 (7.7) | 32.7 (6.4) | 35.2 (7.7) | *p* = .326 |

*: *p* < .05

## Manipulation Check of tVNS

In order to control for the effectiveness of tVNS, we expected heart rate deceleration in the VNS group compared to the control groups during stimulation. Therefore, we assessed participants’ heart rate during both extinction phases with the Vision Recorder software (Brain Products Inc., Munich, Germany). For data processing Vision Analyzer 2.1 software (Brain Products Inc., Munich, Germany) was used. After applying a 30 Hz high cut-off filter, R-peaks were automatically detected and manually controlled. The continuous heart rate was calculated and exported for E1 and E2.

A repeated measures ANOVA containing the within-subject factor time (E1, E2) and the between-subject factor group (VNS, Sham, control) was calculated. Neither a main effect of time (*F*(1,72) = 0.06, *p* = .804, *ƞ_p_^2^* = .001), nor group (*F*(2,72) = 0.44, *p* = .646, *ƞ_p_^2^* = .012) or an interaction of Time x Group (*F*(2,72) = 0.00, *p* = .996, *ƞ_p_^2^* = .000) was found.

As conclusion, with this data we could not show any change in heart rate due to tVNS. Therefore, it is still not clear whether a relatively short and transcutaneous stimulation of the vagus nerve results in physiological changes in humans. The application of further methods to test the manipulation as discussed are warranted in future studies.

## Rating data


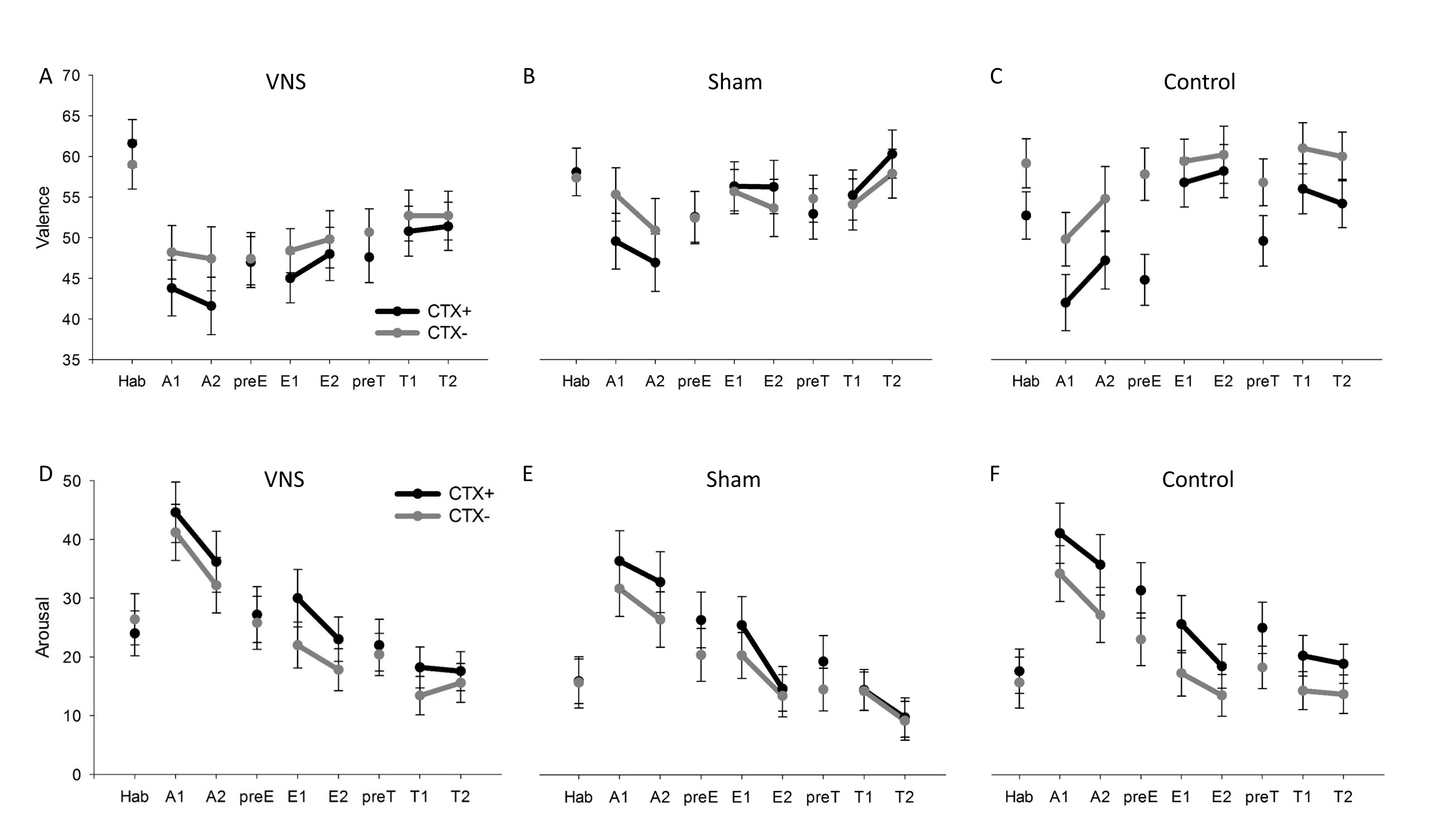

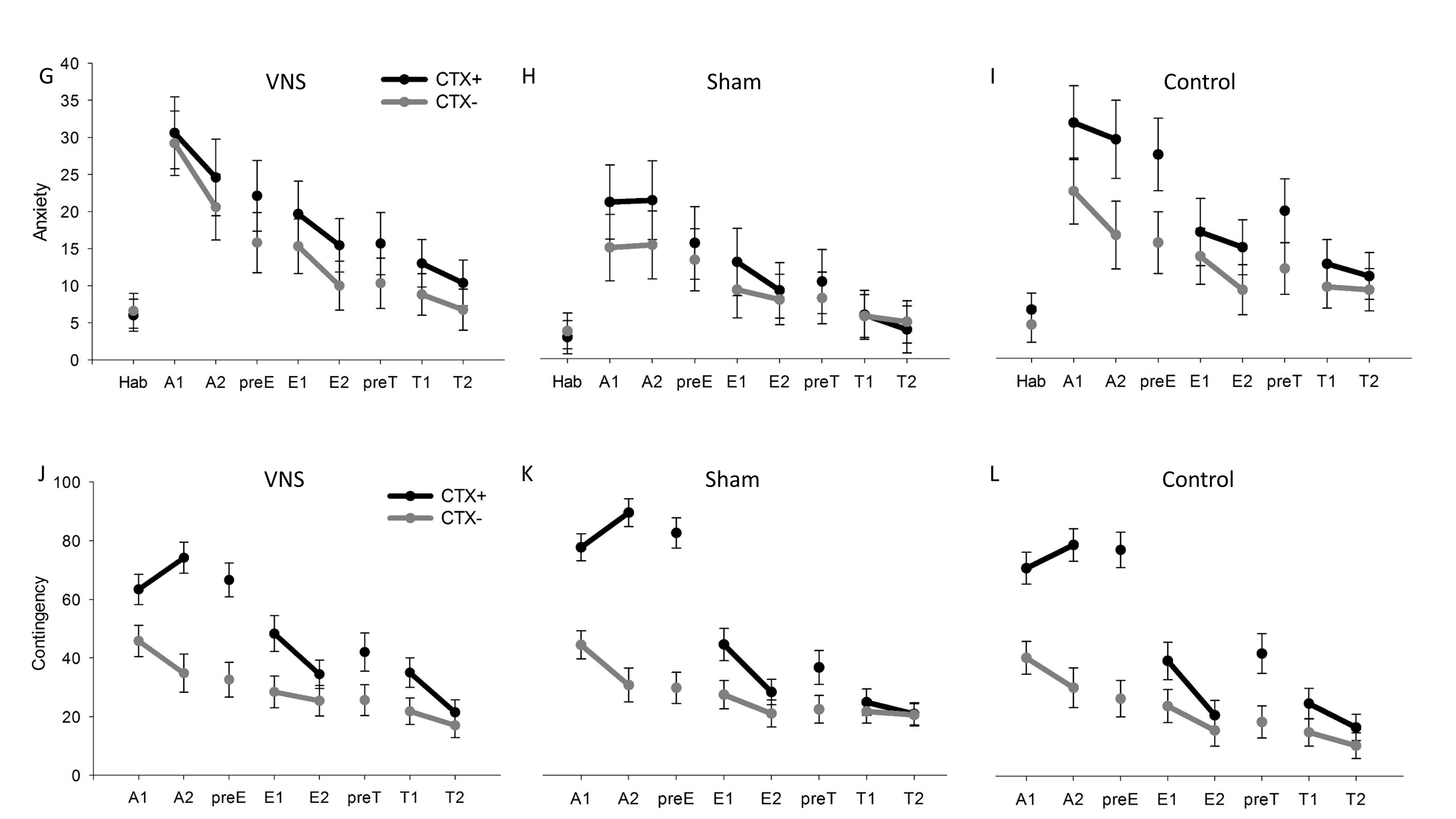


*Suppl. Figure S2:* All ratings separated into groups. Left panel: VNS group (N = 25), middle panel: Sham group (N = 25), right panel: Control group (N = 25). A-C: Valence ratings (0 = very unpleasant, 100 = very pleasant), D-F: Arousal ratings (0 = not arousing, 100 = very arousing), G-H: Anxiety ratings (0 = not anxious, 100 = very anxious), J-L: contingency ratings (0 = surely no US, 100 = surely US). Hab = Habituation, A1 = Acquisition 1, A2 = Acquisition 2, preE = pre-Extinction, E1 = Extinction 1, E2 = Extinction 2, preT = pre-Test, T1 = Test 1, T2 = Test 2.

## Explorative analyses

*Startle and stimulation intensity*. In order to exploratory test stimulation intensity effects on reinstatement of physiological startle response, we calculated difference scores for startle responses between T1 and E2 for CTX+, CTX- and ITI, respectively. Pearson’s correlations between stimulation intensity and differences in startle responses was performed for both stimulated groups (VNS and Sham) separately. The analysis of the VNS group revealed a positive correlation for CTX- (*r*(23) = 0.441, *p* = .028), indicating the higher the stimulation intensity was the greater was the difference of startle amplitude between T1 and E2, which emphasizes greater reinstatement. No significant correlations were found between stimulation intensity and CTX+ (*r*(23) = 0.339, *p* = .098) or ITI (*r*(23) = -0.170, *p* = .415). Correlations in the Sham group were not significant (all *p*s ≥ .488).

*Valence and stimulation intensity*. Furthermore, addressing stimulation intensity effects on reinstatement of valence ratings, we calculated difference scores for valence ratings of preT and E2 for CTX+ and CTX-, respectively. Pearson’s correlations of stimulation intensity and contexts did not correlate with each other, neither in VNS group nor in Sham group (all *p*s ≥ .177).

*Reinstatement and state anxiety.* [Glotzbach-Schoon et al^. 22^](#_ENREF_22) investigated reinstatement dependent on state anxiety. In startle responses, high state anxious participants showed differential reinstatement, whereas low state anxious participants indicated generalized reinstatement. Therefore, they calculated the median split of STAI state (split value was 34) on day 3 just before reinstatement resulting in 11 low anxious (STAI state score: *M* = 30.00, *SD* = 3.00) and 10 high anxious (STAI state score: *M* = 42.40, *SD* = 10.53) participants. Interestingly, for startle responses differential reinstatement was shown in participants with high state anxiety and generalized reinstatement was indicated in participants with low state anxiety. Due to the low number of participants in Glotzbach et al. and the methodological issues on reinstatement like immediate extinction versus extinction and reinstatement on separate days[^43^](#_ENREF_43), replication of these results is needed.

Therefore, we here also calculated a median split of STAI state (split value was 33) on day 3 right before reinstatement ending up with 37 low state anxious participants (*M* = 28.68, *SD* = 3.21) and 38 high state anxious participants (*M* = 40.11, *SD* = 5.52). We calculated an ANOVA with the within-subject factors context (CTX+, CTX-, ITI) and time (E5, T1) and the between-subject factor state anxiety (low state anxiety, high state anxiety). The analyses revealed main effects of context (*F*(2,146) = 19.50, *p* < .001, *ƞ_p_^2^* = .221), phase (*F*(1,73) = 55.57, *p* < .001, *ƞ_p_^2^* = .432) and the interaction of Context x Time (*F*(2,146) = 13.94, *p* < .001, *ƞ_p_^2^* = .160). Additionally, the 3-way interaction Context x Time x State Anxiety turned out to be marginally significant (*F*(2,146) = 2.61, *p* = .077, *ƞ_p_^2^* = .034). Post-hoc *t*-tests for the high and low anxiety group separately revealed no significant differences between CTX+, CTX- and ITI in E5 (all *p*s ≥ .114). However during T5, low anxious participants showed same startle amplitudes for CTX+ and CTX- (*t*(36) = 0.18, *p* = .862) indicating generalized reinstatement, but higher startle amplitudes for CTX+ compared to ITI (*t*(36) = 5.23, *p* < .001) and for CTX- compared to ITI (*t*(36) = 4.63, *p* < .001). In contrast, high anxious participants showed marginally potentiated startle responses in CTX+ compared to CTX- (*t*(37) = 1.77, *p* = .086) indicating differential reinstatement. Similar to the low anxious group, CTX+ (*t*(37) = 4.54, *p* < .001) as well as CTX- (*t*(37) = 3.20, *p* = .003) evoked higher startle responses compared to ITI. These results are in line with the prior study and replicate the state anxiety effect on reinstatement. As it is discussed in [Glotzbach-Schoon et al^. 22^](#_ENREF_22), a mood-congruent memory effect might be an explanation. High anxious participants might remember the emotional content of CTX+ more reliably as it fits to the emotional content of the anxiety memory. In contrast, a positive mood should result in lower reinstatement. However, [Glotzbach-Schoon et al^. 22^](#_ENREF_22) and recent study, both used median splits to separate high and low anxious group. For further investigations, more studies are warranted that specifically manipulate state anxiety.


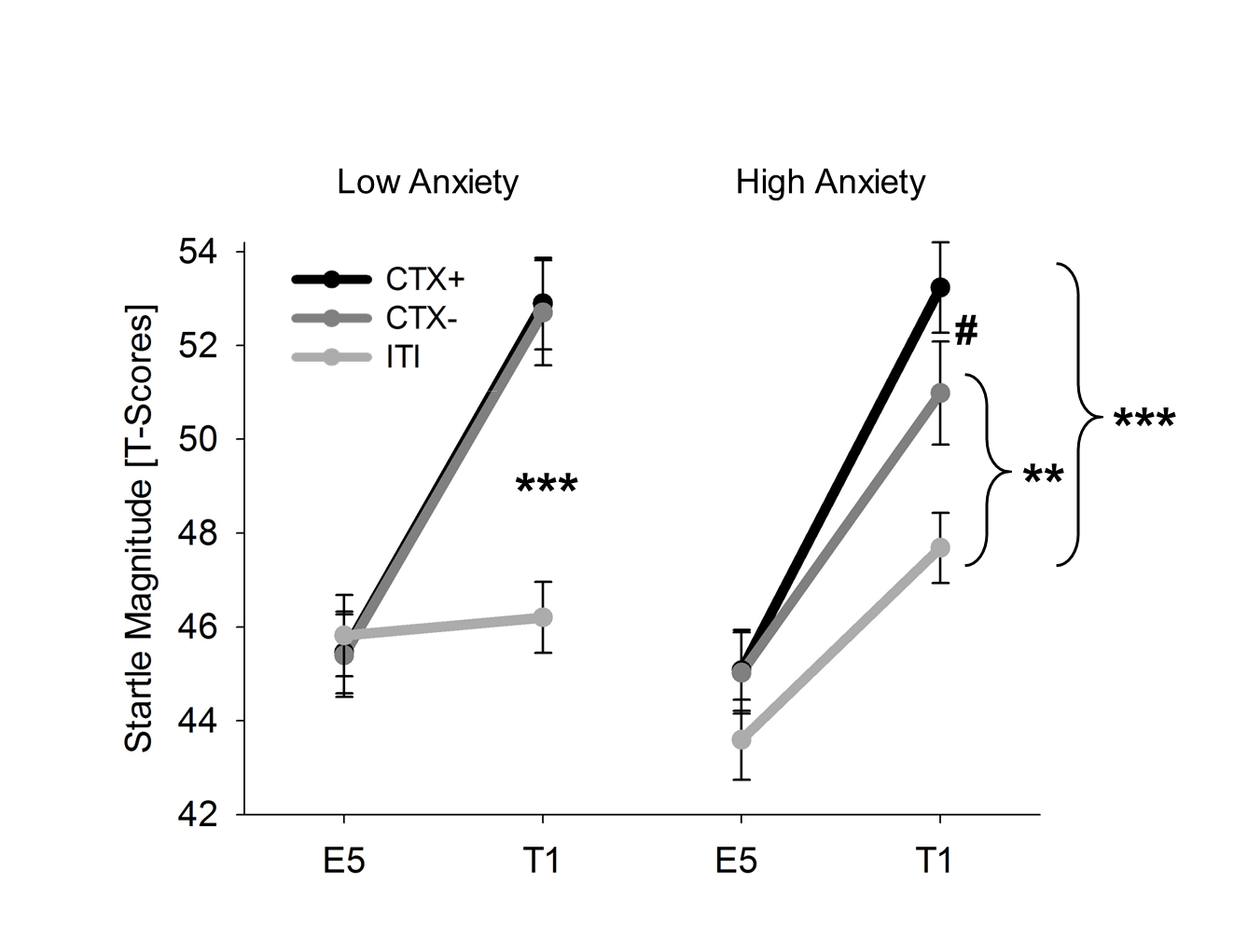


*Suppl. Figure S3*: Reinstatement effects on startle magnitude separated for low (left panel) and high (right panel) state anxious participants. #: *p* < .09; **: *p* < .01; ***: *p* < .001
